# Biomechanics of extreme lateral interbody fusion with different internal fixation methods: a finite element analysis

**DOI:** 10.1186/s12891-022-05049-7

**Published:** 2022-02-09

**Authors:** Xiao-hua Li, Li-jun She, Wei Zhang, Xiao-dong Cheng, Jin-peng Fan

**Affiliations:** 1 Department of Spinal Surgery, The Third Hospital of Hebei Medical University, No. 139 Ziqiang Road, Shijiazhuang, 050051 China; 2 Department of Tuberculosis, The Fifth Hospital of Shijiazhuang, No.42 Tanan Road, Shijiazhuang, 050000 China; 3 Department of Spinal Surgery, The Third Hospital of Hebei Medical University, No. 139 Ziqiang Road, Shijiazhuang, 050051 Hebei Province China; 4 Department of Orthopedic Surgery, Shijiazhuang First Hospital, No. 365 Jianhua South Street, Shijiazhuang, 050000 China

**Keywords:** Extreme lateral interbody fusion, Finite element analysis, Internal fixation, Adjacent segment degeneration, Cage subsidence

## Abstract

**Background:**

Establishing a normal L3–5 model and using finite element analysis to explore the biomechanical characteristics of extreme lateral interbody fusion (XLIF) with different internal fixation methods.

**Method:**

The L3–5 CT image data of a healthy adult male volunteer were selected to establish a normal lumbar finite element model (M0). The range of motion (ROM) of L3–4 and L4–5, under flexion, extension, left bending, right bending, left rotation, and right rotation, together with L3–4 disc pressure was analyzed. Then the L4–5 intervertebral disc was excised and implanted with a cage, supplemented by different types of internal fixation, including lateral two-hole plate model (M1), lateral four-hole plate model (M2), VerteBRIDGE plating model (M3), lateral pedicle model (M4), posterior unilateral pedicle screw model (M5) and posterior bilateral pedicle screw model (M6). The ROM,the maximum stress value of the cage, and the maximum stress value of the intervertebral disc of L3–4 were analyzed and studied .

**Results:**

The ROM of L3–4 and L4-L5 segments in the validation model under various motion states was basically consistent with previous reports. The lumbar finite element model was validated effectively. After XLIF-assisted internal fixation, the range of activity in L3–4 segments of each internal fixation model was greater than that of the normal model under various working conditions, among which the M5、M6 model had the larger range of activity in flexion and extension. After the internal fixation of L4–5 segments, the mobility in M1-M6 was significantly reduced under various motion patterns. In terms of flexion and extension, the posterior pedicle fixation model (M5、M6) showed a significant reduction,followed by M2. The maximal von mises cage stress of M1 was obviously greater than that of other models (except the left bending). Compared with M0, the intervertebral disc stress of M1-M6 at L3–4 segments was increased.

**Conclusions:**

It is recommended that the posterior bilateral pedicle screw model is the first choice, followed by the lateral four-hole plate model for fixation during XLIF surgery. However, it is still necessary to be aware of the occurrence of adjacent segment degeneration (ASD) in the later stage.

## Introduction

Interbody fusion is a classic surgical method for treating degenerative diseases of the lumbar spine. Traditional lumbar interbody fusion involves heavy pulling of soft tissue dissection, dural sac and nerve root, resulting in more postoperative complications. Extreme lateral interbody fusion (XLIF), proposed by Ozgur et al. [1] in 2006, was a surgical method that can be used to gain access to the lumbar spine via a lateral approach that passes through the retroperitoneal fat and psoas major muscle. The advantages of XLIF include no pulling of peripheral tissues such as nerves, blood vessels and peritoneum, and no need to enter the spinal canal for operation, reducing the possibility of injury to the dural sac and cauda equina, effectively avoiding the risks caused by anterior and posterior surgeries, with fewer complications, and extensive clinical application [2–7]. At present, there are few reports on the biomechanics of XLIF internal fixation. Conventional biomechanics tests, such as animal experiments and cadaver specimens tests, cannot fully reflect the real biomechanical changes of the lumbar spine. Moreover, due to the particularity of the human body, the experimental cost is high, and the reproducibility is low. However, the three-dimensional finite element analysis is highly repeatable and can simulate the complex mechanical environment of the human lumbar spine in digital form. It is more vivid, practical and scientific, and is widely used in the field of spine biomechanics. This study aims to use finite element analysis to compare the biomechanical characteristics of XLIF with various internal fixations, and provide a theoretical basis for choosing the best internal fixation scheme.

## Materials and methods

### Design

Three-dimensional finite element analysis test.

### Time and place

From March 2020 to June 2021, it was completed at the Institute of Orthopedic Biomechanics, the Third Hospital of Hebei Medical University.

### Research object

A healthy adult male volunteer, who is 30 years old, 178 cm tall, and 72 kg, was selected. He had no history of low back pain and lower extremity pain, and his physical examination was normal. Routine X-ray, CT and MRI examinations showed no obvious spinal lesions, deformities, tumors, injuries, etc., and no adjacent segment disc degeneration and facet joint hypertrophy. The study design was approved by the institutional review board of the third Hospital of Hebei Medical University before data collection and analysis. The volunteer agreed to the trial protocol and informed consent was obtained from him.

### CT data acquisition

A 64-slice spiral CT from Siemens of Germany was used to continuously scan the L3–5 segment with a thickness of 1.0 mm, and the tomographic image was obtained and saved in the standard DICOM format.

### Construction of the normal model

The saved CT image file in DICOM format was imported into Mimics 14.01 software. First, bone tissue was processed by threshold segmentation, and surrounding tissue structure and editing mask were removed in parallel. Then 3D reconstruction was carried out to preliminarily build the L3–5 3D model, which was stored in STL format. The STL format model is imported into Geomagic 2013 software (Geomagic, Research Triangle Park NC, USA) and saved as an STP solid model through deburring and smooth processing. After that, the solid model is imported into NX 12.0 software (Siemens Product Lifecycle Management Software Inc., USA), and 3D models of intervertebral disc and articular cartilage were established by stretching and Boolean operation commands. Finally, the models obtained in NX were successively imported into Hypermesh14.0 (Altair Company, USA) software in STP format to divide the grid; C3D10M mesh type was used in the vertebral body, and C3D20 mesh type was used in the nucleus pulposus. Seven kinds of ligament models: anterior longitudinal ligament (ALL), posterior longitudinal ligament (PLL), ligamentum flavum (LF), interspinous ligament (ISL), supraspinous ligament (SSL), intertransverse ligament (ITL), and capsular ligament (CL), were constructed, according to the actual structure of the human anatomy atlas [8]. The ligament models were established by truss unit. The surface-to-surface contact element was used to simulate the articular surface. The friction coefficient between the articular surfaces was set to 0.1, and the strength of the joint capsule ligament was to 200 N/mm. Both the bony structure and the intervertebral disc were assumed to be isotropic elastic materials, which were described by the two parameters of elastic modulus and Poisson’s ratio. Ligament was a material that can only withstand tensile loads and had a zero response under compression. The material properties were quoted from literature [9–14], as shown in Table 1.

**Table 1:** Properties of material in the lumbar spine finite element models

Component	Young’s modulus (MPa)	Poisson’s ratio
Cortical bone	12,000	0.3
Cancellous bone	100	0.2
Posterior element	3500	0.25
Endplate	1000	0.4
Annulus	4.2	0.45
Nucleus pulposus	1.0	0.4999
Articular cartilage	10	0.4
ALL	7.8	0.3
PLL	10	0.3
LF	15	0.3
ISL	10	0.3
SSL	8	0.3
ITL	10	0.3
CL	7.5	0.3
PEEK cage	4000	0.3
Implants Plate/Screws/ rods	110,000	0.3

### Validation of normal lumbar spine finite element

**Fig. 1:**
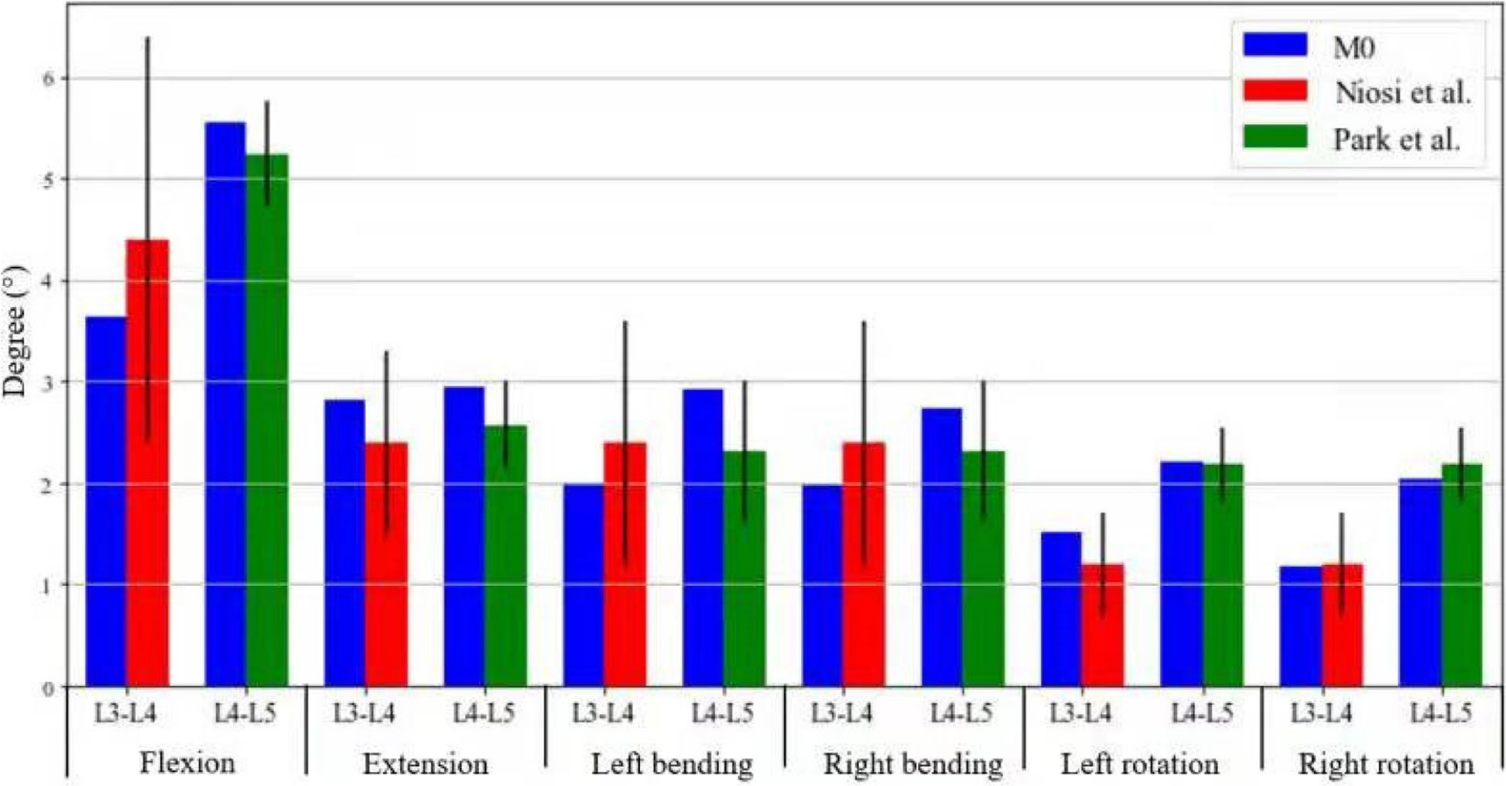
Comparison of the L3–4 ROM between M0 and Niosi et al., L4–5 ROM between M0 and Park et al

First, the weight of the upper body of the human body was simulated; the lower edge of the L5 vertebral body was fixed; and a 7.5 N-m [15] moment with a compressive preload of 500 N was imposed on the superior surfaces of the L3 vertebral body. Then, we calculated the range of motion (ROM) of the L3–4, L4–5 in flexion, extension, left bending, right bending, left rotation, right rotation and L3–4 disc stress. The results showed that the activities of normal lumbar finite element model(M0) and Niosi et al [16] in the L3–4 segment in flexion, extension, left bending, right bending, left rotation, and right rotation were 3.64, 2.82, 1.99, 1.98, 1.51, 1.17 and 4.4 ± 2.0, 2.4 ± 0.9, 2.4 ± 1.2, 2.4 ± 1.2, 1.2 ± 0.5, 1.2 ± 0.5, respectively. In the L4–5 segment, they were 5.54, 2.95, 2.91, 2.74, 2.21, 2.03 and 5.23 ± 0.53, 2.57 ± 0.43, 2.31 ± 0.69, 2.31 ± 0.69, 2.18 ± 0.37, 2.18 ± 0.37 respectively, compared to Park et al. [17] Therefore, the ROM of the L3–4 and L4–5 segments in the model was basically consistent with that reported by Niosi et al. and Park et al., and it was all within a standard deviation range (Fig. 1). This proves that the model is valid and can be used for further experimental research.

### Establishment of XLIF finite element model

**Fig. 2:**
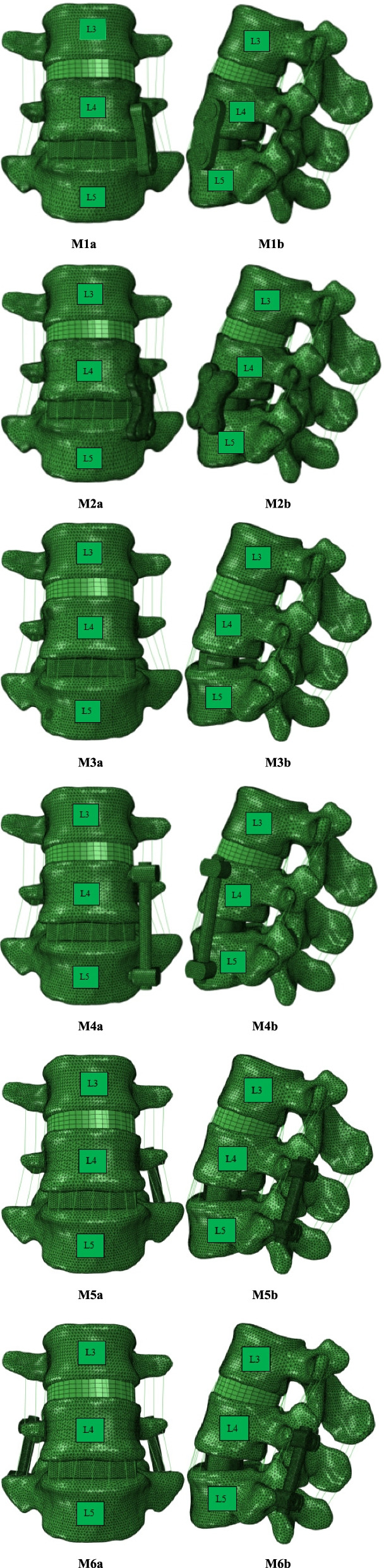
M1-M6 models:M1a-b (lateral two-hole plate model), M2 a-b (lateral four-hole plate model), M3 a-b (VerteBRIDGE plating model), M4a-b (lateral pedicle model), M5a-b (posterior unilateral pedicle screw model), M6a-b (posterior bilateral pedicle screw model)

In NX 12.0 software (Siemens Product Lifecycle Management Software Inc., the United States), the screw rod internal fixation system model, the two-hole plate internal fixation model, the four-hole plate internal fixation model, VerteBRIDGE plating model and the intervertebral fusion cage model were drawn and established, and then optimized and meshed. After that, its position was adjusted by simulating the implantation direction of the steel plate and screw during the operation. Finally it was imported into Ansys to complete the establishment of the finite element model. The contact relationship between the intervertebral implant and the upper and lower endplates was set as surface-to-surface contact method to simulate the pre-fusion state, and the friction coefficient was set as 0.2 [18]. The Sextant system was simulated for pedicle screw fixation with a diameter of 6.5 mm and a length of 50 mm. According to the research content, the models were named as M1 (lateral two-hole plate model), M2 (lateral four-hole plate model), M3 (VerteBRIDGE plating model), M4 (lateral pedicle model), M5 (posterior unilateral pedicle screw model), M6(posterior bilateral pedicle screw model) (see Fig. 2). We implanted the cage model from the left side parallel to the endplate at the level of the L4–5 intervertebral space. M1: Placed the two-hole plate model on the left side of the L4–5 vertebral body, and fixed the two screws at the level of the L4 and L5 vertebral bodies respectively. M2: Placed the four-hole plate model on the left side of the L4–5 vertebral body, and two screws were respectively implanted at the level of the L4 and L5 vertebral bodies. M3: Placed on the left side of the L4–5 vertebral body, and VerteBRIDGE plating model were placed in the L4 and L5 vertebral bodies respectively. M4:Placed 1 pedicle screw on the left side of the L4 and L5 vertebral respectively, and then fixed them with rods. M5: One pedicle screw was implanted in each of the L4 and L5 vertebrae at the level of the lateral edge of the left upper articular process of L4 and L5 along the pedicle direction, and then fixed them with rods.. In the M6, two pedicle screws were implanted into the L4 and L5 vertebrae from both sides according to the same method, and then fixed them with rods. The M1-M4 models were all operated from the left cage implantation incision. M5 and M6 needed to be implanted from the rear and set to be percutaneously fixed without damaging the posterior muscle-ligament complex and articular processes. The contact surface between the internal fixation system and the spine was set to be completely fixed, that is, looseness, displacement, breakage, etc. of the internal fixation were not considered. The lower edge of the L5 vertebral body was fixed to simulate the weight of the upper body of the human body, 500 N pressure was applied to the upper edge of the L3 vertebral body, and 7.5 N.m torque was applied. The ROM of the above model was performed at L3–4 and L4–5 segments, including flexion, extension, left bending, right bending, left rotation, and right rotation. Data for maximum stress of intervertebral cage and maximum pressure of L3–4 intervertebral discs were collected and analyzed.

## Results

### L3–4 rom

**Fig. 3:**
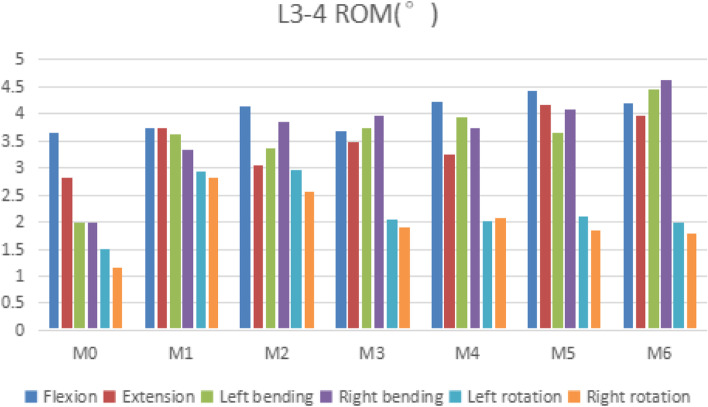
Comparison of the ROM for the L3–4 for seven types of models

Compared with M0, the ROM of the L3–4 segment of M1-M6 was increased under all motion patterns. Compared with M0, M1, M2, and M6 had significant differences in L3–4 ROM, with T values of 3.181, 2.795, and 2.267, respectively, *P* < 0.05, which was statistically significant. But compared with M0, although M3, M4, and M5 had greater mobility in all directions than M0, there was no significant difference in ROM, *P* > 0.05. In terms of flexion, compared with M0, M1-M6 increased by 2.5, 14.0, 0.8, 16.2, 21.4, 15.7%, respectively. In terms of extension, compared with M0, M1-M6 increased by 32.6, 7.8, 23.8, 15.6, 47.9, 41.1%, respectively. Generally speaking, M5 and M6 (posterior pedicle fixation) had relatively high mobility in flexion and extension. M1 and M2 had the largest degree of movement in the left and right rotation, and compared with M0, they had increased by 94.7, 140.2 and 96.7%, and 117.9% respectively. As shown in Fig. 3.

### L4–5 rom

**Fig. 4:**
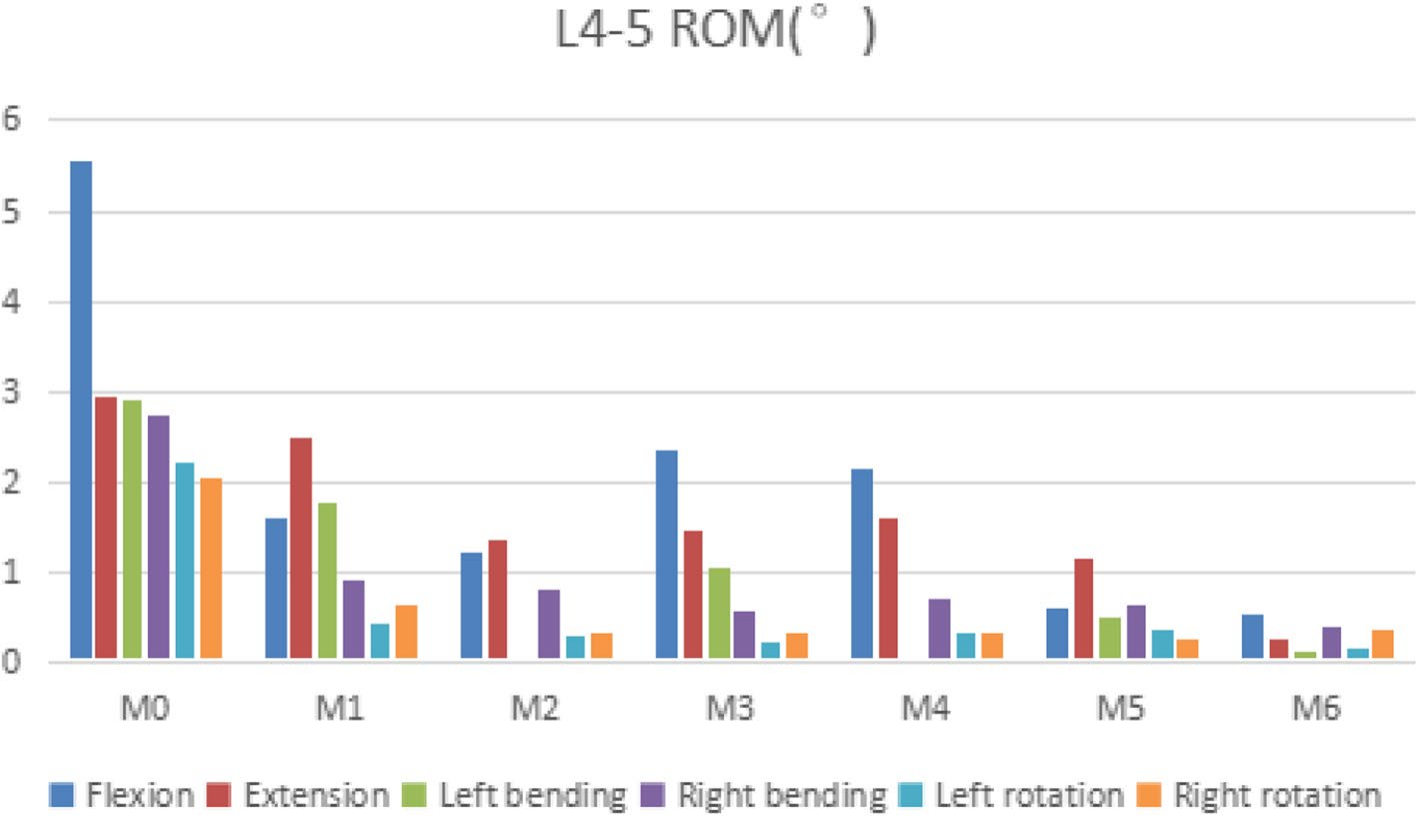
Comparison of the ROM for the L4–5 for seven types of models

After internal fixation, the ROM of M1-M6 in the L4–5 segment was significantly reduced under all motion patterns. Compared with M0, M1-M6 had significant differences in L4–5 ROM, with T values of 3.379, 4.995, 3.922, 3.332, 5.541, 6.315, *P* < 0.05, which was statistically significant. Compared with M0, in terms of flexion and extension, M6 had the most significant decrease, with a decrease of 90.3 and 91.2% respectively. Followed by M5, with a decrease of 89.4 and 61.4% respectively. In the lateral fixed model, M2 decreased by 78.1 and 53.6% in flexion and extension, respectively. In terms of extension, the reduction in M1 was only 15.9%. Compared with M0, the left bending of M2 and M4 was reduced by about 99.3% (see Fig. 4).

### Maximal von Mises stress of the cage

**Fig. 5:**
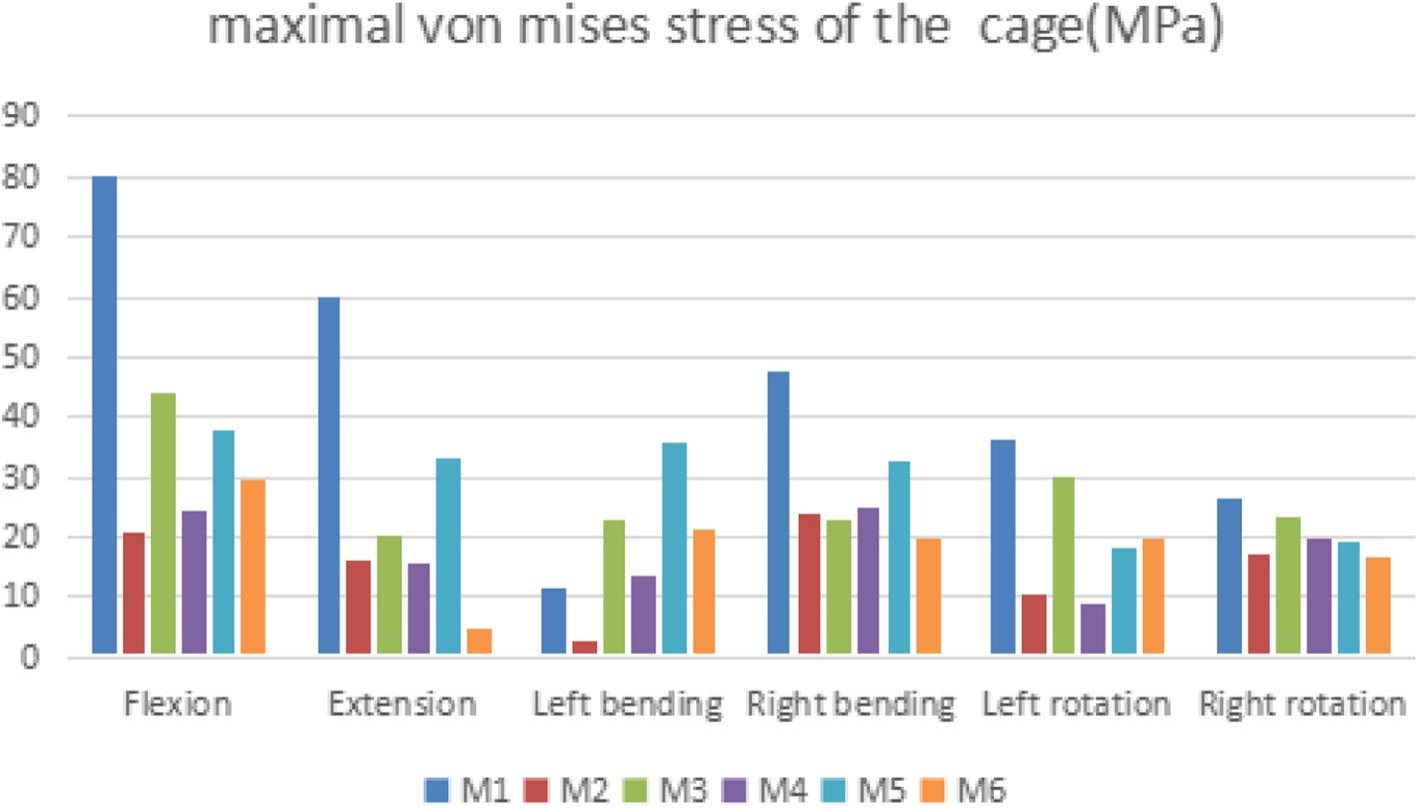
Comparison of the maximal von mises stress of the cage for six types of models

As shown in Fig. 5, the maximal von mises stress of the cage was obviously higher in M1 than that of other models (except for left bending). The stresses of M3 and M5 were also relatively high. Compared with M2, M4, and M6, M1 cage stress increased by 285.3, 226.7, and 168.8% in flexion, respectively. Compared with M2, M4, and M6, the extension of M1 increased by 267.5, 288.5, and 1193.5%, respectively. In the left bending, the stress of M2, M1, and M4 were lower than other models.

### Stress of the L3–4 disc

**Fig. 6:**
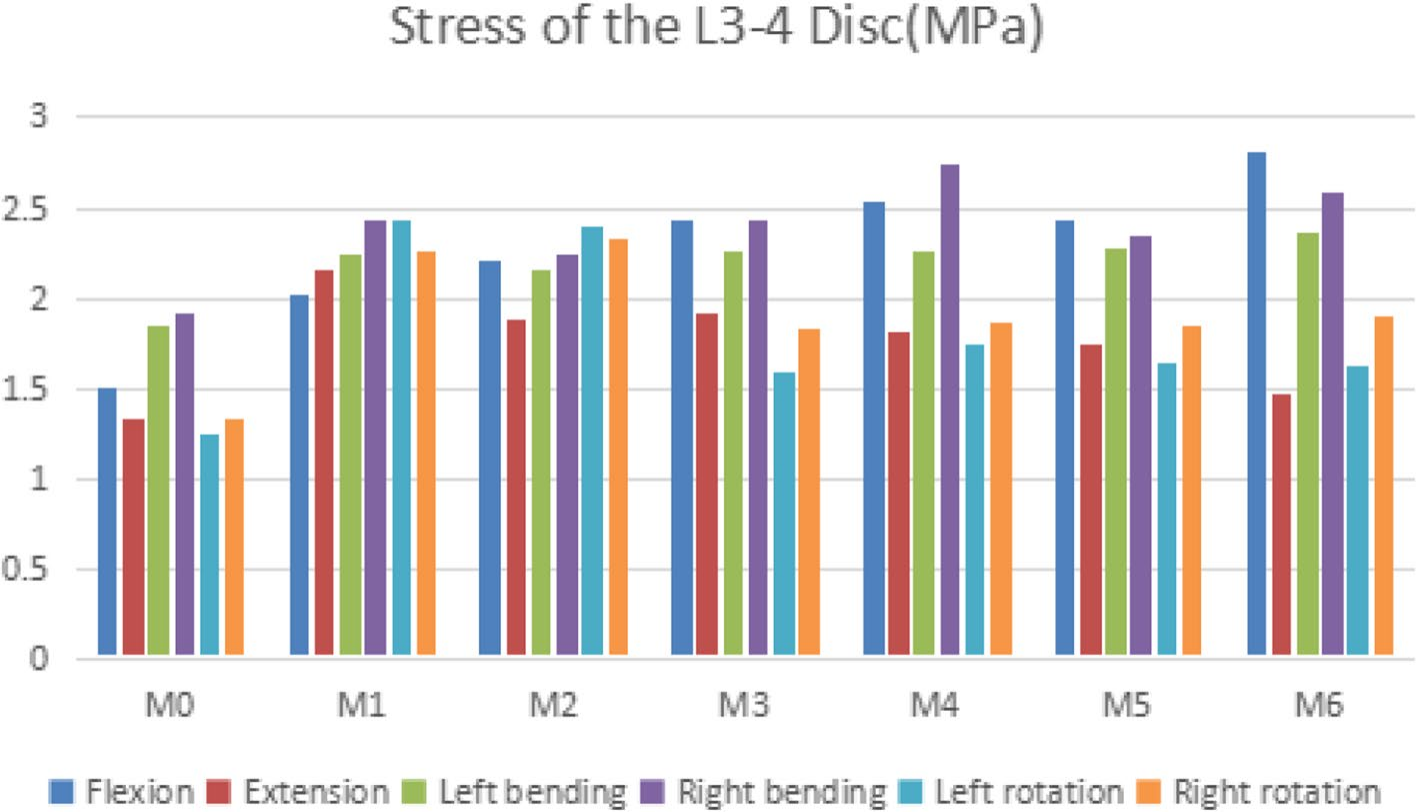
Comparison of the Stress of the L3–4 Disc for seven types of models

As can be seen from Fig. 6 (as follows). Compared with M0, the maximum stress of the L3–4 disc in M1-M6 was found to be higher under all loading conditions. In terms of flexion, compared with M0, M1-M6 increased by 34.6, 47.9, 63.0, 69.9, 62.9, and 87.3%, respectively. In terms of extension, compared with M0, M1-M6 increased by 63.3, 41.6, 30.7, 36.5, 32.1, 11.5%, respectively. In the flexion, M6 had the greatest stress. The stress of M1 was the highest in the extension. M1 and M2 had the greatest stress in left and right rotation.

## Discussion

Finite element analysis is widely used in spinal biomechanics. At present, it is of great significance in the evaluation and analysis of spinal fusion and ASD [19–22]. In the past few years, XLIF was recognized and adopted by spinal surgeons worldwide. Although this operation has many advantages, the complications caused by it should not be ignored. Therefore, improving the stability of the fusion segment, reducing the incidence of cage subsidence and ASD is still the focus of future research.

After lumbar interbody fusion, the biomechanics of the entire spine will change. Its stress load transmission will also change accordingly. In order to stabilize the fusion segment, internal fixation is often supplemented. Clinical studies had shown that, although stand-alone cage can increase the stability of the segment, compared with the additional plate and pedicle screw fixation, the mobility of the fusion segment was significantly increased [23]. .Through imaging analysis, Marchi et al. [24] found that the rate of cage subsidence of stand-alone during XLIF was as high as 30%. Thus, supplementary fixation, such as pedicle screws, is recommended. Studies had shown that lumbar fixation and fusion can significantly reduce the ROM at the fusion level and provide strong stability [25]. Through research in this article, after internal fixation, the ROM of M1-M6 in the L4–5 segment was significantly reduced under all motion patterns. Compared with M0, M1-M6 had significant differences in L4–5 ROM, with T values of 3.379, 4.995, 3.922, 3.332, 5.541, 6.315, *P* < 0.05, which was statistically significant. In terms of flexion and extension, the posterior pedicle fixation model (M5、M6) had a significant decrease, followed by M2, while M1, M3, and M4 had relatively low decreases. It showed that all fixation models could reduce the mobility of the fusion segment. The posterior pedicle fixation model provided high stability, followed by M2, while M1, M3, and M4 were relatively low.

Although much literature reported that there was no significant difference in stability and fusion rate between unilateral and bilateral pedicle fixation [26, 27] many scholars still believed that the strength of unilateral fixation was not as stable as bilateral fixation [28, 29]. Flexion and extension are the most frequent movements in daily human life. In this study, compared with other models, the pedicle screw fixation groups could significantly reduce the mobility of the fusion segment in terms of flexion and extension, indicating that the posterior pedicle fixation group can ensure the stability of the fusion segment, followed by the M2.

Cage subsidence is one of the common complications of lateral lumbar interbody fusion. Macki et al. [30] retrospectively analyzed 21 articles and included 1362 patients undergoing lateral lumbar interbody fusion, and identified that a subsidence incidence of 10.3% and a reoperation rate for subsidence of 2.7%. The maximum stress could be used to predict the sinking risk of the cage. The greater the stress, the higher the sinking risk [13, 31]. Insufficient internal fixation strength was one of the important factors for cage subsidence. The use of supplemental internal fixation for lateral lumbar interbody fusion, such as bilateral pedicle screws, served to mitigate subsidence, protect the indirect decompression, and promote arthrodesis [32]. After fusion, the frictional contact between the cage and the endplate was likely to cause stress concentration. Compared with the unilateral fixation of pedicle screws, the bilateral pedicle screws can effectively control the stability of the index segment and reduce the load applied to the interbody fusion cage. In addition, bilateral fixation had relatively little stress damage to the fusion segment, and the protection of the cage itself was also relatively beneficial [33, 34].

As shown in Fig. 5, the maximal von mises stress of the cage was obviously higher in M1 than that of other models (except for left bending), and the stresses of M3 and M5 were also relatively high. In the left bending, the stress of M2, M1, and M4 were lower than in other models. This shows that M1 has a higher risk of cage subsidence, and the lateral internal fixation device can effectively share the stress of the cage during lateral bending.

After lumbar spine fixation and fusion, the mobility of the fixed segment decreases, causing the center of rotation to shift, thereby changing the motion of adjacent segments. Therefore, the loss of segmental mobility at the fused segments tended to be compensated for by the unfused segments above the fusion [35, 36]. Numerous in vitro experiments have also shown increased mobility of the adjacent segment, presumably as a result of the transfer of motion from the fused segment to the adjacent segment [37–42].

In our study, after internal fixation, the ROM and disc stress of each model in the L3–4 segment in all motion cases were greater than that of the normal model, whereas the M5、M6 model had the larger range of activity in flexion and extension, M6 had a high ROM in left and right bending, and M1 and M2 had the largest ROM in left and right rotation. By analyzing from the stress of the L3/4 disc, it could be concluded that the M6 disc had the highest stress in flexion; M1 had the highest stress in extension; M1 and M2 had the highest stress in left and right rotation. Compared with M0, M3, M4, and M5 had greater mobility in all directions than M0, but in terms of ROM, the difference was not obvious, which may be related to the smaller sample size. Since it is known that rigid fixation causes hypermobility at the adjacent segment, and that hypermobility in turn causes acceleration of the degenerative process [43]. In vitro investigations involving finite element models and human cadaveric spine studies have shown that the pressure of the intervertebral discs in adjacent segments increases after fusion [44, 45]. The biomechanical cause of increased intervertebral disc pressure (IDP) may be that the stiffness of the fusion segment and displaced center of rotation change the kinematics of the adjacent segment caused by lumbar fusion, and the pathological reason of ASD after lumber fusion is that the increased IDP alters the metabolic production and exchange of disc substances [46]. With the increase in the stress of the intervertebral disc in the adjacent segment, the intervertebral disc deforms, resulting in an increase in the mobility of the adjacent segment. Therefore, the increase in the mobility of the adjacent segment may be caused by the increase in the intervertebral disc stress. Increased ROM and intervertebral disc stress in adjacent segments after spinal internal fixation were considered to be the main risk factors for ASD [47, 48], and ASD was considered to be the result of spinal fusion [49].

Overall, all internal fixation models increase the mobility of adjacent segments and the stress of the intervertebral disc. Although the increase in mobility and intervertebral disc stress was not proportional to the strength of internal fixation, ASD should be considered when performing XLIF surgery. Clinical studies had found that the posterior muscle-ligament complex retained during the operation acts as a posterior tension band after the fusion, which could prevent the occurrence of ASD after the fusion to a certain extent [50–52].

Our study was based on finite element analysis and has several limitations. First of all, the division of elements and the determination of boundary conditions in the modeling process were all artificial settings, which need to be compared with cadaver specimens and in vivo experiments. Secondly, the adult healthy volunteer was selected, degenerative factors were not considered, and the muscles were not modeled. Therefore, the force of the human body could not be fully simulated. XLIF is mostly used to treat patients with lumbar degeneration, and factors such as intervertebral disc degeneration, articular process degeneration, and osteoporosis may affect the surgical method. Therefore, the choice of the specific surgical method needs to be combined with the patient’s symptoms and imaging findings. Furthermore, the biomechanical effects of internal fixation and surgery was simplified.

## Conclusion

In conclusion, all fixation models could reduce the mobility of the fusion segment. The posterior pedicle fixation model provided high stability, followed by the lateral four-hole steel plate model. All fixation models could increase the ROM and intervertebral disc stress in adjacent segments. The stress of the cage in lateral two-hole plate model was the largest, followed by VerteBRIDGE plating model and posterior unilateral pedicle screw model. Therefore, it is recommended that the posterior bilateral pedicle screw model is the first choice, followed by the lateral four-hole plate model for fixation during XLIF surgery. However, it is still necessary to be aware of the occurrence of adjacent segment degeneration in the later stage.

## Data Availability

The datasets used and/or analyzed during the current study are available from the corresponding author upon reasonable request.

## Abbreviations

XLIF: Extreme lateral interbody fusion
ROM: Range of motion
ASD: Adjacent segment degeneration
ALL: Anterior longitudinal ligament
PLL: Posterior longitudinal ligament
LF: Ligamentum flavum
ISL: Interspinous ligament
SSL: Supraspinous ligament
ITL: Intertransverse ligament
CL: Capsular ligament
